# Prognostic Value of the Total Bilirubin‐to‐Albumin Ratio in Critically Ill Patients with Gastrointestinal Bleeding: A Multicentre Cohort Study

**DOI:** 10.1155/emmi/1396680

**Published:** 2026-07-25

**Authors:** Xuyong Chen, Shasha Ying, Xiangshu Yuan, Lihong Lv, Xingyi Yang

**Affiliations:** ^1^ Department of Gastroenterology Disease, Xianju People’s Hospital, Zhejiang Southeast Campus of Zhejiang Provincial People’s Hospital, Affiliated Xianju’s Hospital, Hangzhou Medical College, Xianju, Taizhou, Zhejiang, China, hznu.edu.cn; ^2^ Department of Respiratory Medicine, Xianju People’s Hospital, Zhejiang Southeast Campus of Zhejiang Provincial People’s Hospital, Affiliated Xianju’s Hospital, Hangzhou Medical College, Xianju, Taizhou, Zhejiang, China, hznu.edu.cn; ^3^ Department of Laboratory Medicine, Xianju People’s Hospital, Zhejiang Southeast Campus of Zhejiang Provincial People’s Hospital, Affiliated Xianju’s Hospital, Hangzhou Medical College, Xianju, Taizhou, Zhejiang, China, hznu.edu.cn

**Keywords:** gastrointestinal bleeding, mortality, prognosis, total bilirubin-to-serum albumin ratio

## Abstract

**Introduction:**

The total bilirubin‐to‐serum albumin ratio (TBAR) has been proposed as an indicator of hepatic dysfunction and systemic inflammation, yet its prognostic relevance in critically ill patients with gastrointestinal bleeding (GIB) remains unclear. This study evaluated the association between TBAR and mortality and developed a TBAR‐based prognostic model.

**Methods:**

Data were extracted from the MIMIC‐IV v3.1 database, with external validation using the eICU‐CRD v2.0. Adult patients with GIB were included after applying predefined eligibility criteria, yielding a final cohort of 1627 individuals. The primary outcomes were 28‐day all‐cause mortality. Cox regression, Kaplan–Meier analyses, restricted cubic splines (RCS), ROC curves, and subgroup analyses were performed. A prediction nomogram was constructed using variables selected through the Boruta algorithm and evaluated using AUC, C‐index, calibration performance, and decision curve analysis.

**Results:**

Nonsurvivors exhibited markedly greater physiological instability, more severe organ dysfunction, and substantially higher TBAR levels. TBAR was independently associated with mortality across all Cox models (fully adjusted HR for 28‐day mortality: 1.10, 95% CI 1.06–1.15). High TBAR was associated with more than a twofold increased risk of death. TBAR demonstrated superior discriminatory ability compared with TBIL or ALB alone and provided incremental predictive value when combined with the SOFA score. RCS analysis supported a linear dose–response association. Subgroup analyses showed no significant interactions. The final nomogram—which incorporated SOFA, APTT, TBAR, vasopressor use, CRRT, and anion gap—achieved an AUC of 0.794 and a bootstrap‐corrected C‐index of 0.789. External validation in the eICU cohort confirmed the robustness of the association and the strong performance of the model (AUC 0.805).

**Conclusions:**

TBAR is an independent predictor of mortality in critically ill patients with GIB. The TBAR‐based nomogram demonstrates reliable discrimination and calibration and may facilitate individualized mortality risk stratification in clinical practice.

## 1. Introduction

Critically ill gastrointestinal bleeding (GIB) is a common, high‐risk emergency in intensive care, associated with considerable morbidity, mortality, and health‐care resource consumption [1, 2]. From an epidemiological perspective, upper gastrointestinal bleeding (UGIB) accounts for a large proportion of acute GIB, with an estimated annual incidence of 80–150 per 100,000 population and an overall mortality of 2%–10% in general cohorts [3]. In contrast, lower gastrointestinal bleeding (LGIB) is less frequent in the general population, with an estimated annual incidence of 205 per 100,000 (approximately one‐fifth that of UGIB) and an overall mortality of 2%–4% [4, 5]. At the health‐system level, GIB and related gastrointestinal disorders impose a substantial burden on emergency department visits, hospital admissions, and health‐care expenditure [6]. However, once GIB progresses to require ICU management, prognosis often deteriorates markedly and commonly coexists with hemodynamic instability, coagulopathy, and evolving multiorgan dysfunction [1, 7, 8]. These features underscore the importance of early risk stratification to enable timely intervention and to optimize allocation of ICU resources.

Elevated total bilirubin may reflect tissue hypoperfusion related to ischemia [9] and may also indicate increased hepatic metabolic burden following massive gastrointestinal hemorrhage [10]; higher bilirubin has been associated with poor prognosis in patients with GIB [11, 12]. By contrast, serum albumin integrates information on systemic inflammatory burden, nutritional reserve, and hepatic synthetic function [13, 14]. In peptic ulcer bleeding, hypoalbuminaemia has been linked to increased risks of mortality and rebleeding [15]. Therefore, the total bilirubin‐to‐albumin ratio (TBAR) may capture, more comprehensively than either biomarker alone, the combined effects of hepatic dysfunction and systemic inflammation or malnutrition. TBAR has shown prognostic discrimination in other high‐risk populations, including acute pancreatitis and cirrhosis [16, 17].

Despite growing interest in bilirubin–albumin‐based indices, evidence specific to TBAR in critically ill patients with GIB—who are typically more complex and clinically heterogeneous—remains limited, and large ICU‐based evaluations are scarce. We therefore assessed the prognostic value of TBAR in critically ill patients with GIB using the MIMIC‐IV database and validated the findings in an independent multicentre cohort from the eICU‐CRD [18, 19].

## 2. Methods

### 2.1. Database Description

This study utilized data from MIMIC‐IV (version 3.1), a publicly accessible critical care database developed through a collaboration between the Massachusetts Institute of Technology and Beth Israel Deaconess Medical Center. MIMIC‐IV contains deidentified clinical information for all ICU admissions from 2008 to 2022, including demographic characteristics, vital signs, laboratory measurements, comorbidities, therapeutic interventions, and clinical outcomes. To assess the generalizability of our findings, we additionally employed the eICU‐CRD (version 2.0) as an external validation cohort. The eICU‐CRD aggregates anonymized clinical data from more than 200,000 ICU encounters spanning 208 U.S. hospitals between 2014 and 2015.

The first author obtained access upon completion of the required CITI programme (Certification ID: 67058598). As the datasets contain only deidentified information, institutional review board approval and patient informed consent were waived.

### 2.2. Patient Selection

**FIGURE 1:**
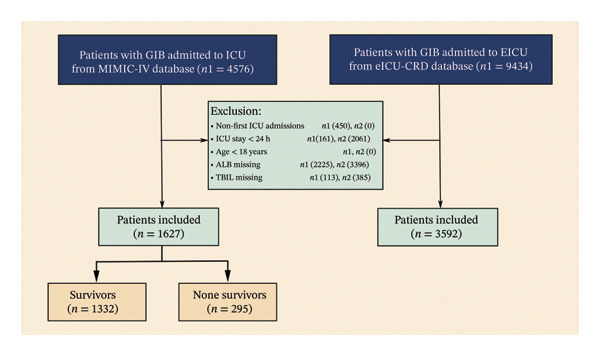
Flow diagram of study participants.

Patients with GIB were identified using International Classification of Diseases, Ninth and Tenth Revision (ICD‐9 and ICD‐10) diagnostic codes in the MIMIC‐IV database. A total of 4576 GIB‐related ICU admissions were initially screened. We excluded patients younger than 18 years (*n* = 0), those without a first ICU admission (*n* = 450), ICU stays of less than 24 h (*n* = 161), and individuals missing essential laboratory variables, including serum albumin (ALB; *n* = 2225) or total bilirubin (TBIL; *n* = 113). Overall, 2949 of 4576 initially screened admissions (64.4%) were excluded, primarily because ALB measurements were unavailable within the first 24 h after ICU admission. Because TBAR requires both ALB and TBIL for calculation, patients missing either component could not be included in the primary exposure analysis. After applying all inclusion and exclusion criteria, 1627 adult patients with GIB were retained for the final analytical cohort (Figure 1). To further characterize the missingness pattern of the exposure‐defining laboratory variables, we compared baseline characteristics between patients included in the primary analysis and those excluded because ALB or TBIL was unavailable within the first 24 h of ICU admission and to assess potential selection bias related to laboratory availability.

### 2.3. Data Collection

We extracted six principal categories of clinical information from the database: (1) demographic characteristics, (2) vital signs, (3) laboratory measurements, (4) comorbidities, (5) severity scores, and (6) therapeutic interventions and outcomes. All laboratory variables were calculated using mean values obtained within the first 24 h after ICU admission. The core exposure variable was the total bilirubin‐to‐serum albumin ratio (TBAR). The primary outcome was 28‐day all‐cause mortality, and the secondary outcome was 90‐day all‐cause mortality. A comprehensive list of baseline variables is provided in Supporting Table 1. These available variables were also used to provide clinical context regarding physiological instability, organ dysfunction, and management intensity in critically ill patients with GIB. Detailed clinical and procedural information directly related to GIB severity, including bleeding source, endoscopic findings, transfusion volume, timing of endoscopy, and endoscopic hemostatic procedures, was not consistently available in structured fields across the MIMIC‐IV and eICU‐CRD databases and therefore could not be incorporated into the primary models. To address this limitation quantitatively as far as possible, we included available indicators reflecting physiological instability, organ dysfunction, coagulation abnormality, and treatment intensity, including heart rate, respiratory rate, blood pressure, APTT, BUN, SOFA score, acute kidney injury (AKI), cirrhosis, sepsis, vasopressor use, mechanical ventilation (MV), and continuous renal replacement therapy (CRRT) use.

### 2.4. Statistical Analysis

Normally distributed continuous variables are presented as mean ± SD and compared with Student′s *t*‐test; non‐normal variables as median (IQR) and compared with the Wilcoxon rank‐sum test. Categorical variables are reported as *n* (%) and compared using the *χ* ^2^ or Fisher’s exact test, as appropriate.

Because TBAR and other covariates were derived from data obtained during the first 24 h after ICU admission, the analytical cohort was restricted to patients with an ICU stay of at least 24 h. The first 24 h after ICU admission were therefore regarded as the exposure‐assessment window, during which TBAR and other baseline covariates were defined. Patients who did not complete this 24‐h window were excluded before analysis. Accordingly, the survival and prediction analyses should be interpreted within a 24‐h landmark framework among patients who completed the initial exposure‐assessment window. This design helped ensure that exposures and adjustment covariates were defined before risk prediction and helped reduce potential time‐dependent bias. X‐tile software was used to determine the optimal cut‐off value for TBAR. Covariates were selected based on the results of LASSO regression combined with variables considered clinically important (Supporting Figures 1–3). In this study, LASSO regression was used only to assist in selecting adjustment covariates for the association analyses, whereas the Boruta algorithm was used separately to identify robust predictors for nomogram development. Using these inputs, we constructed three Cox models with increasing levels of adjustment. Model 1 included age, sex, and race; Model 2 added heart rate, respiratory rate, red blood cell count, and anion gap (AG); and Model 3 further incorporated APTT, BUN, SOFA score, AKI, cirrhosis, diabetes, sepsis, CRRT, MV, and vasopressor use. Multicollinearity between variables included in Cox Model 3 and the final nomogram was assessed using variance inflation factors (VIFs), with VIF values < 5 considered to indicate no substantial multicollinearity. Kaplan–Meier curves with log‐rank tests were used to compare survival across TBAR categories.

The predictive performance of TBAR for 28‐day mortality was evaluated using receiver operating characteristic (ROC) curves, while restricted cubic spline (RCS) modeling was used to examine potential nonlinear associations between TBAR and mortality risk. A nomogram incorporating the six predictors selected by the Boruta algorithm was subsequently developed to predict 28‐day mortality, and model performance was assessed using the concordance index (C‐index), calibration curves, area under the ROC curve (AUC), and decision curve analysis (DCA). For clinical interpretability, the nomogram‐derived predicted probabilities were categorized into three pragmatic risk groups: low risk (< 10%), intermediate risk (10%–20%), and high risk (≥ 20%). These thresholds were selected to distinguish patients with predicted risks below, around, and above the overall 28‐day mortality rate of the cohort, and were intended to support bedside risk stratification rather than serve as absolute treatment thresholds.

To further assess the robustness and generalizability of our findings, correlation analyses and independent external validation of the prediction model were conducted using the eICU‐CRD.

Subgroup analyses stratified by age, gender, race, AKI, cirrhosis, diabetes mellitus (DM), myocardial infarction (MI), ischemic heart disease (IHD), chronic obstructive pulmonary disease (COPD), CRRT, MV, and vasopressor use were performed to evaluate potential effect modification.

Multiple imputation by chained equations (10 imputations; mice package in R) was applied to variables with < 20% missingness, while variables with ≥ 20% missing data were excluded. In addition, to assess the robustness of the findings to potential selection bias related to laboratory availability, we performed a sensitivity analysis using multiple imputation for missing ALB and TBIL. Statistical analyses were conducted using R (version 4.4.1). This study was reported in accordance with the Transparent Reporting of a multivariable prediction model for Individual Prognosis or Diagnosis (TRIPOD) statement. A completed TRIPOD checklist for prediction model development and validation is provided as a supporting file.

## 3. Results

### 3.1. Baseline Characteristics

A total of 1627 patients were included, of whom 295 (18.1%) died within 28 days of ICU admission. As shown in Table 1, nonsurvivors exhibited more pronounced physiological instability, with significantly higher heart and respiratory rates and lower blood pressure (all *p* < 0.001). Laboratory profiles also differed markedly between groups: nonsurvivors had worse hepatic and renal function, higher inflammatory and coagulation abnormalities, and substantially elevated TBAR levels (all *p* < 0.05, most *p* < 0.001). Furthermore, nonsurvivors had higher SOFA scores (*p* < 0.001) and a greater prevalence of AKI, cirrhosis, heart failure, MI, and sepsis (all *p* < 0.05). The use of vasopressors, MV, and CRRT was also significantly more frequent among nonsurvivors (all *p* < 0.001). Taken together, these available clinical variables suggest that nonsurvivors had greater physiological instability, more severe coagulation and organ dysfunction, and higher treatment intensity, providing additional context for bleeding severity and clinical management.

**TABLE 1:** Patient demographics and baseline characteristics.

Variables	All patients (*n* = 1627)	Survivors (*n* = 1332)	Nonsurvivors (*n* = 295)	*p* value
*Demographic*				
Age, years	65 (55–77)	65 (55–77)	66 (55–77)	0.478
Gender, *n* (%)				0.409
Male	1016 (62.4%)	838 (62.9%)	178 (60.3%)	
Female	611 (37.6%)	494 (37.1%)	117 (39.7%)	
Race, *n* (%)				0.047
White	1052 (64.7%)	876 (65.8%)	176 (59.7%)	
Other	575 (35.3%)	456 (34.2%)	119 (40.3%)	

*Vital signs*				
HR, beats/min	88 (76–99)	87 (75–98)	92 (81–104)	< 0.001
SBP, mmHg	112 (103–125)	113 (104–126)	107 (101–117)	< 0.001
DBP, mmHg	62 (56–69)	62 (56–70)	59 (53–68)	< 0.001
MAP, mmHg	75 (68–83)	75 (69–83)	72 (66–79)	< 0.001
RR, breaths/min	18.5 (16.3–21.5)	18.3 (16.2–21.1)	19.8 (17.4–23.3)	< 0.001
Temperature, °C	36.78 (36.57–37.04)	36.79 (36.59–37.05)	36.70 (36.45–36.98)	< 0.001

*Laboratory indicators*				
Hematocrit, %	27.4 (24.5–31.0)	27.5 (24.6–31.1)	27.1 (23.8–30.5)	0.065
Hemoglobin, g/dL	9.08 (8.00–10.38)	9.11 (8.03–10.47)	8.93 (7.83–10.02)	0.043
Platelet, 10^9^/L	142 (86–217)	147 (91–218)	117 (69–215)	< 0.001
RDW, %	16.33 (14.90–18.30)	16.20 (14.80–18.08)	17.20 (15.53–19.53)	< 0.001
RBC, 10^12^/L	3.01 (2.63–3.46)	3.06 (2.67–3.48)	2.86 (2.52–3.32)	< 0.001
WBC, 10^9^/L	10 (7–15)	10 (7–14)	13 (9–19)	< 0.001
ALB, g/dL	2.90 (2.50–3.30)	2.95 (2.60–3.30)	2.80 (2.40–3.30)	< 0.001
AG, mEq/L	14.0 (11.5–17.2)	13.7 (11.3–16.6)	16.3 (13.0–19.5)	< 0.001
TCa, mg/dL	8.07 (7.60–8.57)	8.05 (7.60–8.50)	8.17 (7.67–8.87)	0.004
Chloride, mEq/L	105 (100–109)	105 (100–109)	102 (97–108)	< 0.001
Glucose, mg/dL	128 (106–162)	126 (105–159)	137 (111–174)	< 0.001
Potassium, mEq/L	4.20 (3.80–4.65)	4.15 (3.80–4.60)	4.30 (3.90–4.80)	< 0.001
Sodium, mEq/L	138.5 (135.0–141.5)	138.8 (135.3–141.5)	137.3 (133.0–141.5)	< 0.001
INR	1.45 (1.20–1.80)	1.40 (1.20–1.73)	1.70 (1.35–2.25)	< 0.001
PT, s	16 (14–20)	15 (13–19)	19 (15–24)	< 0.001
APTT, s	33 (28–42)	32 (28–39)	42 (33–57)	< 0.001
ALT, IU/L	26 (15–54)	24 (15–47)	38 (20–79)	< 0.001
AST, IU/L	44 (23–102)	40 (22–86)	77 (37–171)	< 0.001
TBIL, mg/dL	1.1 (0.5–3.0)	1.0 (0.5–2.4)	2.2 (0.7–8.8)	< 0.001
Creatinine, mg/dL	1.23 (0.80–2.20)	1.15 (0.80–2.00)	1.78 (1.10–3.00)	< 0.001
BUN, mg/dL	32 (19–53)	30 (18–50)	41 (26–67)	< 0.001
SOFA	6.0 (4.0–10.0)	6.0 (3.0–9.0)	10.0 (7.0–13.0)	< 0.001
TBAR	0.38 (0.18–1.07)	0.35 (0.17–0.84)	0.87 (0.26–3.23)	< 0.001

*Comorbidity*				
HTN, *n* (%)	530 (32.6%)	437 (32.8%)	93 (31.5%)	0.671
AKI, *n* (%)	862 (53.0%)	630 (47.3%)	232 (78.6%)	< 0.001
Cirrhosis, *n* (%)	557 (34.2%)	420 (31.5%)	137 (46.4%)	< 0.001
Diabetes, *n* (%)	469 (28.8%)	386 (29.0%)	83 (28.1%)	0.772
HF, *n* (%)	482 (29.6%)	378 (28.4%)	104 (35.3%)	0.019
MI, *n* (%)	135 (8.3%)	102 (7.7%)	33 (11.2%)	0.047
IHD, *n* (%)	510 (31.3%)	416 (31.2%)	94 (31.9%)	0.832
COPD, *n* (%)	220 (13.5%)	175 (13.1%)	45 (15.3%)	0.336
Sepsis, *n* (%)	1101 (67.7%)	842 (63.2%)	259 (87.8%)	< 0.001

*Treatment*				
CRRT, *n* (%)	193 (11.9%)	116 (8.7%)	77 (26.1%)	< 0.001
MV, *n* (%)	1281 (78.7%)	1020 (76.6%)	261 (88.5%)	< 0.001
Vasopressors, *n* (%)	851 (52.3%)	607 (45.6%)	244 (82.7%)	< 0.001

*Group*				
TBAR (≤ 2.05), *n* (%)	1384 (85.1%)	1189 (89.3%)	195 (66.1%)	< 0.001
TBAR (> 2.05), *n* (%)	243 (14.9%)	143 (10.7%)	100 (33.9%)	

*Note:* Continuous variables are presented as median (interquartile range, 25th–75th percentile), and categorical variables as *n* (%). Between‐group comparisons were performed using the Mann–Whitney *U* test for continuous variables and the chi‐square test or Fisher’s exact test for categorical variables, as appropriate. AST, aspartate aminotransferase; ALT, alanine aminotransferase; ALB, albumin; RDW, red cell distribution width; TBIL, total bilirubin.

Abbreviations: AG, anion gap; AKI, acute kidney injury; BUN, blood urea nitrogen; CRRT, continuous renal replacement therapy; DBP, diastolic blood pressure; HF, heart failure; HR, heart rate; IHD, ischemic heart disease; INR, international normalized ratio; MAP, mean arterial pressure; MI, myocardial infarction; MV, mechanical ventilation; RBC, red blood cell; RR, respiratory rate; SBP, systolic blood pressure; SOFA, Sequential Organ Failure Assessment; TBAR, total bilirubin‐to‐albumin ratio; WBC, white blood cell.

To characterize the missingness pattern, we compared patients included in the primary analysis with those excluded because ALB or TBIL was unavailable within the first 24 h of ICU admission (Supporting Table 2). Patients with available ALB and TBIL measurements differed substantially from excluded patients and generally represented a higher‐acuity subgroup, with greater illness severity, more frequent organ support, and higher 28‐day and 90‐day mortality. These findings suggest that laboratory availability was associated with clinical severity and that missingness was unlikely to be completely random. Therefore, the final analytical cohort may represent a subgroup of critically ill patients with GIB who underwent early liver‐related laboratory assessment, rather than the entire spectrum of ICU patients with GIB. However, the association between TBAR and mortality remained significant in the multiple‐imputation sensitivity analysis, supporting the robustness of the primary findings.

In sensitivity analyses based on multiple imputation for missing ALB and TBIL (Supporting Table 3), the association between TBAR and both 28‐day and 90‐day mortality remained statistically significant across all models, supporting the robustness of the primary findings.

### 3.2. Cox Regression Analysis

**FIGURE 2:**
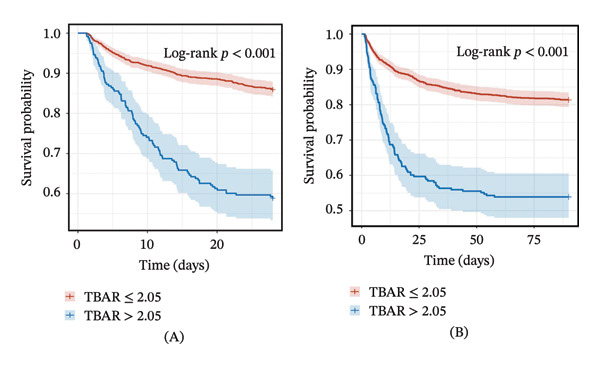
K–M survival curves for all‐cause mortality at 28‐day (A) and 90‐day (B).

Higher TBAR levels were consistently and independently associated with increased mortality across all Cox models. When analyzed as a continuous variable, TBAR showed a strong association with 28‐day mortality in Model 1 (HR 1.21, 95% CI 1.16–1.23), and this association persisted in Model 2 (HR 1.16, 95% CI 1.12–1.20) and Model 3 (HR 1.10, 95% CI 1.06–1.15), all *p* < 0.001. Similar patterns were observed for 90‐day mortality, with HRs of 1.18, 1.15, and 1.10 across the three models (all *p* < 0.001). In the binary classification analysis, patients in the high‐TBAR group had markedly higher mortality risks, with Model 3 HRs of 2.16 (95% CI 1.59–2.93) for 28‐day mortality and 2.00 (95% CI 1.52–2.64) for 90‐day mortality (both *p* < 0.001). These results indicate that the prognostic effect of TBAR remained robust across multiple levels of adjustment (Table 2). To address potential multicollinearity between TBAR and SOFA, we calculated VIF values for variables included in Cox Model 3. The VIF values for TBAR and SOFA were 1.44 and 2.24, respectively, and all variables had VIF values below 5, suggesting no substantial multicollinearity. Kaplan–Meier survival curves showed that patients with higher TBAR levels had significantly lower survival probabilities at both 28 and 90 days. The high‐TBAR group exhibited a consistently reduced survival rate compared with the low‐TBAR group (log‐rank *p* < 0.001), as illustrated in Figure 2.

**TABLE 2:** Association between TBAR and mortality in gastrointestinal bleeding.

Variable	Model 1		Model 2		Model 3	
	HR (95% CI)	*p* value	HR (95% CI)	*p* value	HR (95% CI)	*p* value
*28-day mortality*						
TBAR (continuous)	1.21 (1.16–1.23)	< 0.001	1.16 (1.12–1.20)	< 0.001	1.10 (1.06–1.15)	< 0.001

*TBAR (categorical)*						
Low TBAR	1		1		1	
High TBAR	4.40 (3.38–5.74)	< 0.001	3.70 (2.79–4.90)	< 0.001	2.16 (1.59–2.93)	< 0.001

*90-day mortality*						
TBAR (continuous)	1.18 (1.15–1.22)	< 0.001	1.15 (1.11–1.18)	< 0.001	1.10 (1.06–1.14)	< 0.001

*TBAR (categorical)*						
Low TBAR	1		1		1	
High TBAR	3.71 (2.91–4.72)	< 0.001	3.22 (2.49–4.16)	< 0.001	2.00 (1.52–2.64)	< 0.001

*Note:* Model 1: adjusted for age, gender, and race. Model 2: adjusted for age, gender, race, HR, RR, RBC, and AG. Model 3: adjusted for age, gender, race, HR, RR, RBC, AG, APTT, BUN, SOFA, AKI, cirrhosis, diabetes mellitus, sepsis, CRRT, MV, and vasopressors.

Abbreviations: CI, confidence interval; HR, hazard ratio; TBAR, total bilirubin‐to‐albumin ratio.

### 3.3. Predictive Performance of TBAR and Combined Models

**FIGURE 3:**
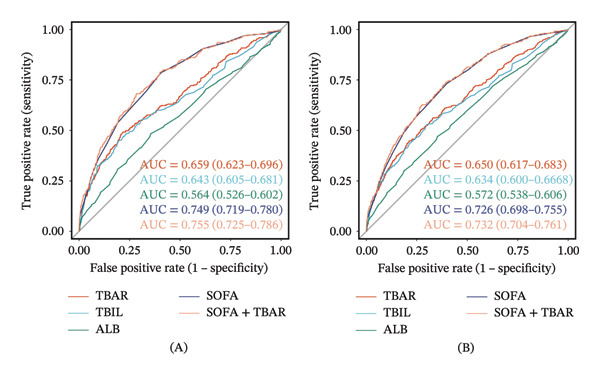
ROC analysis for 28‐day (A) and 90‐day (B) mortality.

We next evaluated the predictive performance of TBAR, TBIL, ALB, and the SOFA score using ROC analysis. TBIL showed a moderate ability to predict mortality, whereas TBAR outperformed both TBIL and ALB in DeLong tests. For both 28‐day and 90‐day mortality, TBAR had significantly higher discriminative ability than TBIL and ALB, with all comparison‐specific DeLong *p* values < 0.001. The ROC curves are presented in Figure 3, and the detailed ROC metrics and AUC values are shown in Table 3. In addition, combining TBAR with the SOFA score improved its predictive performance, and this improvement was consistent for both 28‐day and 90‐day mortality.

**TABLE 3:** Information of ROC curves in Figure 3.

Variable	AUC	95% CI	Threshold	Sensitivity	Specificity	DeLong *p* value vs. TBAR
*28-day mortality*						
TBAR	0.659	0.623–0.696	0.986	0.492	0.784	Reference
TBIL	0.643	0.605–0.681	3.200	0.447	0.805	< 0.001
ALB	0.564	0.526–0.602	2.750	0.485	0.644	< 0.001
SOFA	0.749	0.719–0.780	7.000	0.786	0.594	N/A
SOFA + TBAR	0.755	0.725–0.786	N/A	0.186	0.969	N/A

*90-day mortality*						
TBAR	0.650	0.617–0.683	0.985	0.449	0.788	Reference
TBIL	0.634	0.600–0.668	1.900	0.530	0.700	< 0.001
ALB	0.572	0.538–0.606	2.750	0.470	0.648	< 0.001
SOFA	0.726	0.698–0.755	7.000	0.732	0.601	N/A
SOFA + TBAR	0.732	0.704–0.761	N/A	0.186	0.955	N/A

*Note:* TBIL, total bilirubin; ALB, albumin.

Abbreviations: AUC, area under the curve; CI, confidence interval; ROC, receiver operating characteristic; SOFA, Sequential Organ Failure Assessment; TBAR, total bilirubin‐to‐albumin ratio.

### 3.4. Evaluation of Linearity Between TBAR and Mortality Risk

**FIGURE 4:**
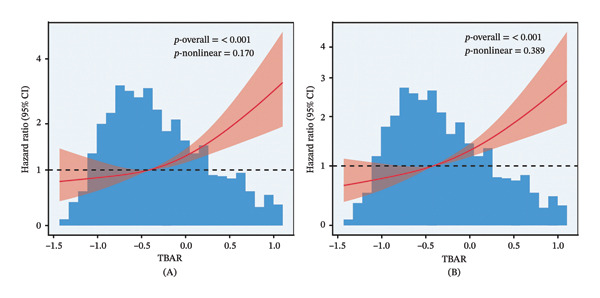
Restricted cubic spline curves illustrating the association between the TBAR and mortality at 28‐day (A) and 90‐day (B) follow‐up.

We further examined the potential nonlinear association between TBAR and short‐ and mid‐term mortality in critically ill patients with GIB using RCS analysis. The spline curves indicated an overall linear pattern, with mortality risk increasing steadily as TBAR levels rose. This was supported by a nonsignificant test for nonlinearity (*p* for nonlinearity > 0.05), as shown in Figure 4.

### 3.5. Subgroup Analyses

**FIGURE 5:**
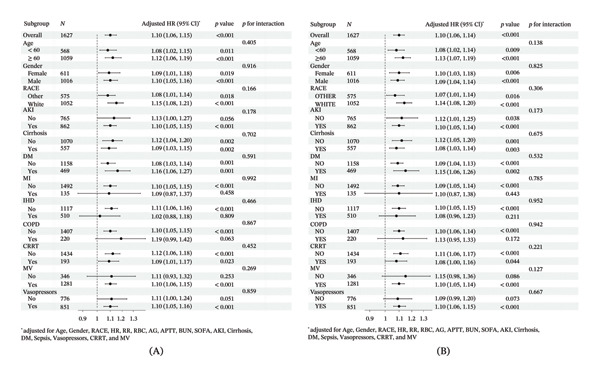
Subgroup analysis of the association between TBAR and (A) 28‐day and (B) 90‐day mortality.

To further assess the robustness of the association between TBAR and mortality, we performed subgroup analyses across several clinically relevant strata, including age, gender, race, AKI, cirrhosis, DM, MI, IHD, COPD, CRRT, MV, and vasopressor use (Figure 5).

### 3.6. Model Development and Validation

**FIGURE 6:**
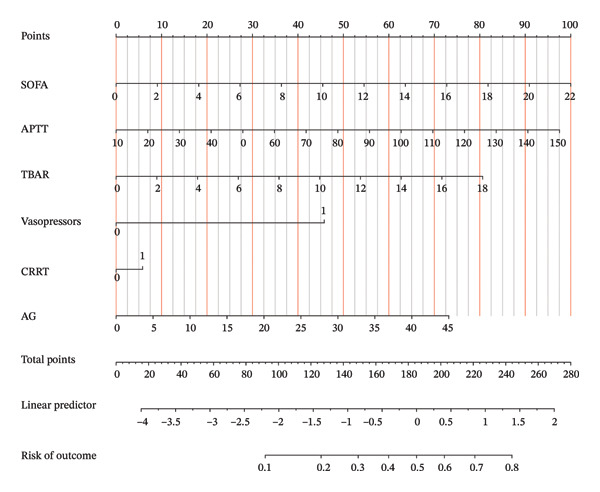
Nomogram for predicting 28‐day mortality in critically ill patients with gastrointestinal bleeding.

Using the Boruta feature selection algorithm, we identified the six most influential predictors of 28‐day mortality—SOFA score, APTT, TBAR, vasopressor use, CRRT use, and AG—as shown in Supporting Figure 4. These variables were incorporated to construct a mortality prediction nomogram tailored for patients with GIB (Figure 6). To facilitate clinical translation, we further developed a web‐based dynamic risk calculator based on this nomogram, which allows clinicians to input individual patient variables and obtain real‐time estimates of 28‐day mortality risk (available at: https://icu-risk-predictor.shinyapps.io/dynnomapp/).

The nomogram demonstrated good discriminatory ability, yielding an AUC of 0.794 (95% CI 0.767–0.821) (Supporting Figure 5). Calibration plots showed close agreement between predicted and observed mortality (Supporting Figure 6). Internal validation using a nonparametric bootstrap procedure with 1000 resamples produced an optimism‐corrected Harrell’s C‐index of 0.789, indicating robust performance and limited overfitting. DCA further showed that the nomogram offered a net clinical benefit across a relatively broad range of threshold probabilities (Supporting Figure 7), supporting its potential usefulness for individualized mortality risk stratification in patients with GIB. According to the nomogram‐derived predicted probabilities, patients were classified into low‐risk (< 10%), intermediate‐risk (10%–20%), and high‐risk (≥ 20%) groups. The observed 28‐day mortality increased progressively across these categories, from 4.3% in the low‐risk group to 17.5% in the intermediate‐risk group and 37.3% in the high‐risk group (*p* < 0.001; Supporting Table 4). These findings support the clinical interpretability of the proposed risk stratification and provide a more intuitive framework for applying the web‐based dynamic calculator in bedside risk assessment.

### 3.7. External Validation

To further ensure the robustness and generalizability of our findings, we performed an external validation using the eICU‐CRD database. The associations between TBAR levels and mortality remained consistent with those observed in the MIMIC‐IV cohort. Cox proportional hazards models confirmed that higher TBAR levels were independently associated with increased mortality across all adjustment levels (HR 1.07–1.18 per unit increase, all *p* < 0.001), with a clear dose–response relationship across quartiles (*p* for trend < 0.001). When the cut‐off of 2.05 was applied in the eICU cohort, elevated TBAR remained independently associated with mortality in the fully adjusted model (HR 1.39, 95% CI 1.04–1.86; *p* = 0.026). Sensitivity analyses using nearby cut‐offs of 1.8 and 2.2 yielded broadly similar results (HR 1.57, 95% CI 1.18–2.09; *p* = 0.002, and HR 1.55, 95% CI 1.17–2.06; *p* = 0.003, respectively), supporting the robustness of the selected cut‐off (Supporting Table 5). K–M curves, RCS analyses, and subgroup findings demonstrated patterns comparable to the derivation cohort (Supporting Figures 8–10). The ROC performance of TBAR in the eICU cohort was also similar to that observed in the MIMIC analysis, further supporting the reproducibility of its discrimination ability.

External validation of the prediction nomogram likewise demonstrated good discrimination and acceptable calibration. Specifically, the fixed nomogram model developed in the MIMIC‐IV cohort was applied to the external eICU‐CRD validation cohort, achieving an AUC of 0.805, a Brier score of 0.0856, a calibration slope of 1.144, and a calibration intercept of 0.357 (Supporting Figure 11). These findings suggest that the prediction model retained good discrimination and acceptable external calibration in the external cohort, although the positive calibration intercept indicated some degree of overall risk underestimation. Overall, these results support the external transportability of TBAR and the proposed prediction model in patients with GIB, while also suggesting that further local recalibration may be needed before broader clinical implementation.

## 4. Discussion

In this study, we found that the TBAR was independently associated with short‐term mortality. This association was robust to extensive adjustment (28‐day mortality: adjusted HR 1.10 per unit increase, 95% CI 1.06–1.15; *p* < 0.001) and remained evident when TBAR was analyzed categorically (high vs low TBAR: adjusted HR 2.16, 95% CI 1.59–2.93). TBAR also showed moderate discrimination and outperformed total bilirubin or albumin alone, while providing incremental prognostic information when combined with the SOFA score. The risk relationship appeared approximately linear on RCS analyses, and prespecified subgroup analyses suggested consistency across clinically relevant strata. Finally, a parsimonious TBAR‐containing nomogram demonstrated good discrimination (AUC 0.794, 95% CI 0.767–0.821) and retained performance on external validation in eICU (AUC 0.805, 95% CI 0.783–0.828), supporting the reproducibility of our findings across cohorts.

Composite bilirubin–albumin indices may enhance prognostic assessment in both acute and chronic severe illness by integrating markers of cholestasis/hepatocellular injury (bilirubin) with hepatic synthetic function, systemic inflammation, and nutritional reserve (albumin). In a retrospective ICU cohort including 477 patients with acute pancreatitis, Yang et al. [16] reported that an elevated TBAR (cut‐off 1.33) was independently associated with increased mortality after multivariable adjustment. Similarly, in 509 critically ill patients with cirrhosis, Yang et al. [17] found that the direct bilirubin‐to‐albumin ratio independently predicted 28‐day mortality, with moderate discrimination. Together, these findings support the inclusion of bilirubin–albumin ratios in routine risk stratification and justify further validation across diverse clinical settings. However, no clinically established TBAR cut‐off has yet been proposed specifically for critically ill patients with GIB. Therefore, the cut‐off of 2.05 identified in the present study should be interpreted in a disease‐specific context and should not be directly extrapolated from thresholds derived in other populations.

From a pathophysiological perspective, hyperbilirubinaemia in critically ill GIB may reflect the combined effects of systemic hypoperfusion and evolving multiorgan dysfunction [20, 21]. Severe hemorrhage can precipitate haemorrhagic shock and tissue hypoxia, impairing hepatic blood flow and bile excretion and contributing to cholestasis and hepatocellular injury [9, 22]. Concomitant systemic inflammation—often amplified by infection or sepsis in the ICU—may further disrupt hepatobiliary transport and metabolism [23, 24], while transfusion‐related hemolysis can increase bilirubin production [25].

In contrast, hypoalbuminaemia is a well‐recognized surrogate of reduced hepatic synthetic capacity, heightened inflammatory burden, and poor nutritional reserve [26]. Lower albumin reduces colloid oncotic pressure, which may exacerbate intravascular volume depletion and capillary leak during acute bleeding [27, 28]. Albumin also reflects physiological reserve; consequently, low concentrations frequently co‐occur with complications such as AKI and nosocomial infection, and are closely associated with poor prognosis [29–31]. Together, elevated bilirubin and reduced albumin capture complementary dimensions of critical illness severity that are biologically coherent with adverse outcomes in severe GIB.

The TBAR integrates two routinely measured biomarkers that capture complementary pathophysiological dimensions in critically ill patients with GIB: hyperbilirubinaemia reflects impaired perfusion and evolving organ dysfunction, whereas hypoalbuminaemia indicates reduced hepatic synthetic capacity, depleted nutritional reserve, and heightened inflammatory burden. By combining these signals, TBAR may provide a pragmatic, multidimensional index of prognosis. Importantly, both components are inexpensive and readily available from standard laboratory testing, making TBAR well suited to early risk stratification in the ICU. Clinically, an elevated TBAR identifies patients at higher risk of adverse outcomes who may warrant closer monitoring and more proactive management, whereas a lower TBAR may support standard care pathways—thereby facilitating rational allocation of critical care resources and timely escalation for those most likely to deteriorate. From an emergency medicine perspective, TBAR may be most useful as an early adjunctive risk‐stratification marker rather than as a standalone decision‐making tool. In patients presenting with GIB, an elevated TBAR may help identify individuals who require closer monitoring, earlier senior clinician review, consideration of ICU admission or escalation of care, and timely consultation for endoscopic or hemostatic evaluation. Because ALB and TBIL are routinely available laboratory tests, TBAR can be rapidly calculated together with conventional assessments of hemodynamic status, bleeding severity, coagulation, and organ dysfunction. However, TBAR should not replace established clinical evaluation, resuscitation assessment, or bleeding‐source‐specific management decisions. Instead, it may provide additional prognostic information to support triage and prioritization in emergency and critical care settings.

Several limitations should be noted. First, given the clinical heterogeneity of GIB, important information on aetiology, severity, and key management (including transfusion) was not fully captured. Detailed data on bleeding source, endoscopic findings, hemostatic procedures, and transfusion volume could not be comprehensively extracted from the databases. The absence of these variables may have introduced residual confounding and may have limited the interpretability and generalizability of the prediction model, because laboratory and organ‐support variables cannot fully capture bleeding‐source‐specific risk or treatment response. Therefore, the association between TBAR and mortality should be interpreted in the context of incomplete bleeding‐specific severity and management information. Second, because more than 60% of initially screened ICU admissions were excluded due to unavailable ALB or TBIL measurements, selection bias related to nonrandom laboratory availability cannot be excluded. This large exclusion was mainly attributable to unavailable ALB measurements within the first 24 h after ICU admission and was methodologically necessary because TBAR could not be calculated without both ALB and TBIL. Patients with these measurements available appeared to represent a more severely ill subgroup undergoing more intensive evaluation, which may limit generalizability to less severely ill patients with GIB. Although multiple‐imputation sensitivity analyses and external validation in the eICU‐CRD cohort supported the robustness of the findings, and therefore strengthened the reliability of the main prognostic association, differences in cohort selection and case mix between the derivation and validation datasets may also have influenced the observed discrimination performance, and residual bias cannot be completely ruled out. Third, the TBAR cut‐off of 2.05 was determined using a data‐driven X‐tile approach in the derivation cohort and may therefore be cohort‐specific. Although this cut‐off was directly applied in the eICU‐CRD external validation cohort and nearby‐threshold sensitivity analyses using 1.8 and 2.2 yielded consistent results, this cut‐off should still be regarded as exploratory rather than a definitive clinical threshold. Finally, both cohorts derive from a single country and health‐care system, so generalizability to other regions and resource settings warrants caution.

## 5. Conclusion

In this multicentre cohort of critically ill patients with GIB, the TBAR was independently associated with short‐term mortality and demonstrated consistent prognostic value across external validation. A TBAR‐based nomogram showed acceptable discrimination and calibration, supporting its potential utility for early risk stratification. Further prospective studies are required to validate these findings and clarify the clinical role of TBAR in routine assessment.

**Nomenclature**
TBAR: Total bilirubin‐to‐serum albumin ratio
GIB: Gastrointestinal bleeding
RCS: Restricted cubic splines
UGIB: Upper gastrointestinal bleeding
ROC: Receiver operating characteristic
C‐index: Concordance index
DCA: Decision curve analysis
APTT: Activated partial thromboplastin time
BUN: Blood urea nitrogen
AKI: Acute kidney injury
DM: Diabetes mellitus
MI: Myocardial infarction
IHD: Ischemic heart disease
COPD: Chronic obstructive pulmonary disease
CRRT: Continuous renal replacement therapy
MV: Mechanical ventilation

## Author Contributions

Xuyong Chen: methodology, formal analysis, data curation, writing–original draft, and visualization.

Shasha Ying: methodology, formal analysis, investigation, and writing–original draft.

Xiangshu Yuan: validation and writing–review and editing.

Lihong Lv: validation.

Xingyi Yang: conceptualization, supervision, project administration, resources, and writing–review and editing.

## Funding

No funding was received for this work.

## Disclosure

All authors approved the final manuscript and concur with the submission.

## Ethics Statement

The MIMIC‐IV database was approved by the Institutional Review Board (IRB) of Beth Israel Deaconess Medical Center (Protocol No. 2001P‐001699/14). The eICU Collaborative Research Database (eICU‐CRD) is a multicentre critical care database that has been deidentified in accordance with the Health Insurance Portability and Accountability Act (HIPAA) Safe Harbor standard and is made available for research use under a data use agreement. In both databases, all personal identifiers were removed and replaced with anonymized codes. As this study is a retrospective secondary analysis of publicly available, fully deidentified data, additional institutional approval was not required and the requirement for individual informed consent was waived.

## Conflicts of Interest

The authors declare no conflicts of interest.

## Supporting Information

Additional supporting information can be found online in the Supporting Information section.

## Supporting information


**Supporting Information 1** Supporting Table 1: Variables extracted from the MIMIC‐IV database. Supporting Table 2: Baseline comparison between included patients and patients excluded because of missing albumin and/or total bilirubin. Supporting Table 3: Sensitivity analysis after multiple imputation for missing serum albumin and total bilirubin: association of TBAR with 28‐day and 90‐day mortality. Supporting Table 4: Risk stratification according to nomogram‐derived predicted probabilities of 28‐day mortality. Supporting Table 5: External validation in the eICU‐CRD cohort. Supporting Figure 1: Ten‐fold cross‐validation for LASSO model tuning. Supporting Figure 2: LASSO coefficient profiles of candidate predictors. Supporting Figure 3: Optimal λ selection and identification of final features. Supporting Figure 4: Boruta‐selected features for 28‐day mortality. Supporting Figure 5: ROC curve showing the discriminative performance of the nomogram for 28‐day mortality. Supporting Figure 6: Calibration plot illustrating the agreement between predicted and observed 28‐day mortality. Supporting Figure 7: Decision curve analysis for 28‐day mortality. Supporting Figure 8: K–M survival curves stratified by TBAR levels in external validation. Supporting Figure 9: External validation ROC curve using the eICU‐CRD cohort. Supporting Figure 10: Restricted cubic spline of TBAR in the eICU‐CRD cohort. Supporting Figure 11: ROC analysis of the nomogram for external validation.

**Supporting Information 2** TRIPOD checklist.

## Data Availability

All data are sourced from https://physionet.org/content/mimiciv/3.1/.

## References

[1] Cook D. J. , Griffith L. E. , Walter S. D. et al., The Attributable Mortality and Length of Intensive Care Unit Stay of Clinically Important Gastrointestinal Bleeding in Critically Ill Patients, Critical Care. (2001) 5, no. 6, 368–375, 10.1186/cc1071. 11737927 PMC83859

[2] Campbell H. E. , Stokes E. A. , Bargo D. et al., Costs and Quality of Life Associated With Acute Upper Gastrointestinal Bleeding in the UK: Cohort Analysis of Patients in a Cluster Randomised Trial, BMJ Open. (2015) 5, no. 4, 10.1136/bmjopen-2014-007230. PMC4420945 25926146

[3] Antunes C. , Tian C. , and Copelin I. I. E. L. , Upper Gastrointestinal Bleeding, 2025, Treasure Island (FL).

[4] Longstreth G. F. , Epidemiology and Outcome of Patients Hospitalized With Acute Lower Gastrointestinal Hemorrhage: A Population-Based Study, American Journal of Gastroenterology. (1997) 92, no. 3, 419–424. 9068461

[5] Strate L. L. , Ayanian J. Z. , Kotler G. , and Syngal S. , Risk Factors for Mortality in Lower Intestinal Bleeding, Clinical Gastroenterology and Hepatology. (2008) 6, no. 9, 1004–1010, 10.1016/j.cgh.2008.03.021. 18558513 PMC2643270

[6] Peery A. F. , Crockett S. D. , Murphy C. C. et al., Burden and Cost of Gastrointestinal, Liver, and Pancreatic Diseases in the United States: Update 2021, Gastroenterology. (2022) 162, no. 2, 621–644, 10.1053/j.gastro.2021.10.017. 34678215 PMC10756322

[7] Krag M. , Perner A. , Wetterslev J. et al., Prevalence and Outcome of Gastrointestinal Bleeding and Use of Acid Suppressants in Acutely Ill Adult Intensive Care Patients, Intensive Care Medicine. (2015) 41, no. 5, 833–845, 10.1007/s00134-015-3725-1. 25860444

[8] Nagesh V. K. , Pulipaka S. P. , Bhuju R. et al., Management of Gastrointestinal Bleed in the Intensive Care Setting, an Updated Literature Review, World Journal of Critical Care Medicine. (2025) 14, no. 1, 10.5492/wjccm.v14.i1.101639. PMC11671843 40060732

[9] Waseem N. and Chen P. , Hypoxic Hepatitis: A Review and Clinical Update, Journal of Clinical and Translational Hepatology. (2016) 4, no. 3, 263–268, 10.14218/JCTH.2016.00022. 27777895 PMC5075010

[10] Roche S. P. and Kobos R. , Jaundice in the Adult Patient, American Family Physician. (2004) 69, no. 2, 299–304. 14765767

[11] Moledina S. M. and Komba E. , Risk Factors for Mortality Among Patients Admitted With Upper Gastrointestinal Bleeding at a Tertiary Hospital: A Prospective Cohort Study, BMC Gastroenterology. (2017) 17, no. 1, 10.1186/s12876-017-0712-8. PMC5738843 29262794

[12] Zou D. , Qi X. , Zhu C. et al., Albumin-Bilirubin Score for Predicting the in-Hospital Mortality of Acute Upper Gastrointestinal Bleeding in Liver Cirrhosis: A Retrospective Study, Turkish Journal of Gastroenterology. (2016) 27, no. 2, 180–186, 10.5152/tjg.2016.15502. 27015623

[13] Soeters P. B. , Wolfe R. R. , and Shenkin A. , Hypoalbuminemia: Pathogenesis and Clinical Significance, JPEN—Journal of Parenteral and Enteral Nutrition. (2019) 43, no. 2, 181–193, 10.1002/jpen.1451. 30288759 PMC7379941

[14] Don B. R. and Kaysen G. , Serum Albumin: Relationship to Inflammation and Nutrition, Seminars in Dialysis. (2004) 17, no. 6, 432–437, 10.1111/j.0894-0959.2004.17603.x. 15660573

[15] Cheng H. , Yang E. , Wu C. et al., Hypoalbuminemia is a Predictor of Mortality and Rebleeding in Peptic Ulcer Bleeding Under Proton Pump Inhibitor Use, Journal of the Formosan Medical Association. (2018) 117, no. 4, 316–325, 10.1016/j.jfma.2017.07.006. 28751088

[16] Yang X. , Zhang M. , Lv L. , Chen X. , and Li Z. , Total Bilirubin-to-Albumin Ratio and Short- and Long-Term All-Cause Mortality in Acute Pancreatitis: Evidence From the MIMIC-IV Database, PLoS One. (2025) 20, no. 5, 10.1371/journal.pone.0323330. PMC12097592 40403080

[17] Yang X. , Wang G. , Min Z. , Lv L. , and Yang J. , The Prognostic Value of the Direct Bilirubin to Albumin Ratio in Critically Ill Patients With Cirrhosis: Insights From MIMIC-IV Database, PLoS One. (2025) 20, no. 10, 10.1371/journal.pone.0334591. PMC12517503 41082514

[18] Johnson A. E. W. , Bulgarelli L. , Shen L. et al., MIMIC-IV, a Freely Accessible Electronic Health Record Dataset, Scientific Data. (2023) 10, no. 1, 10.1038/s41597-022-01899-x. PMC9810617 36596836

[19] Pollard T. J. , Johnson A. E. W. , Raffa J. D. , Celi L. A. , Mark R. G. , and Badawi O. , The eICU Collaborative Research Database, a Freely Available Multi-Center Database for Critical Care Research, Scientific Data. (2018) 5, no. 1, 10.1038/sdata.2018.178. PMC6132188 30204154

[20] Horvatits T. , Trauner M. , and Fuhrmann V. , Hypoxic Liver Injury and Cholestasis in Critically Ill Patients, Current Opinion in Critical Care. (2013) 19, no. 2, 128–132, 10.1097/mcc.0b013e32835ec9e6. 23403733

[21] Kramer L. , Jordan B. , Druml W. , Bauer P. , and Metnitz P. G. H. , Null. Incidence and Prognosis of Early Hepatic Dysfunction in Critically Ill Patients—A Prospective Multicenter Study, Critical Care Medicine. (2007) 35, no. 4, 1099–1104. 17334250 10.1097/01.CCM.0000259462.97164.A0

[22] Nakatani T. , Sakamoto Y. , Ando H. , and Kobayashi K. , Bile and Bilirubin Excretion in Relation to Hepatic Energy Status During Hemorrhagic Shock and Hypoxemia in Rabbits, The Journal of Trauma. (1995) 39, no. 4, 665–670, 10.1097/00005373-199510000-00008. 7473951

[23] Bhogal H. K. and Sanyal A. J. , The Molecular Pathogenesis of Cholestasis in Sepsis, Frontiers in Bioscience. (2013) 5, no. 1, 87–96. 10.2741/e598 PMC4944118 23276972

[24] Green R. M. , Beier D. , and Gollan J. L. , Regulation of Hepatocyte Bile Salt Transporters by Endotoxin and Inflammatory Cytokines in Rodents, Gastroenterology. (1996) 111, no. 1, 193–198, 10.1053/gast.1996.v111.pm8698199. 8698199

[25] L′Acqua C. , Bandyopadhyay S. , Francis R. O. et al., Red Blood Cell Transfusion is Associated With Increased Hemolysis and an Acute Phase Response in a Subset of Critically Ill Children, American Journal of Hematology. (2015) 90, no. 10, 915–920, 10.1002/ajh.24119. 26183122 PMC4831067

[26] Gabay C. and Kushner I. , Acute-Phase Proteins and Other Systemic Responses to Inflammation, New England Journal of Medicine. (1999) 340, no. 6, 448–454, 10.1056/nejm199902113400607. 9971870

[27] van de Wouw J. and Joles J. A. , Albumin is an Interface Between Blood Plasma and Cell Membrane, and Not Just a Sponge, Clinical Kidney Journal. (2022) 15, no. 4, 624–634, 10.1093/ckj/sfab194. 35371452 PMC8967674

[28] Saravi B. , Goebel U. , Hassenzahl L. O. et al., Capillary Leak and Endothelial Permeability in Critically Ill Patients: A Current Overview, Intensive Care Medicine Experimental. (2023) 11, no. 1, 10.1186/s40635-023-00582-8. PMC10733291 38117435

[29] Wiedermann C. J. , Wiedermann W. , and Joannidis M. , Hypoalbuminemia and Acute Kidney Injury: A Meta-Analysis of Observational Clinical Studies, Intensive Care Medicine. (2010) 36, no. 10, 1657–1665, 10.1007/s00134-010-1928-z. 20517593 PMC7728653

[30] Wiedermann C. J. , Hypoalbuminemia as Surrogate and Culprit of Infections, International Journal of Molecular Sciences. (2021) 22, no. 9, 10.3390/ijms22094496. PMC8123513 33925831

[31] de V. E. D. , Mosquera J. M. , Rubio J. J. et al., Association of a Low Serum Albumin with Infection and Increased Mortality in Critically Ill Patients, Intensive Care Medicine. (1980) 7, no. 1, 19–22. 7451716 10.1007/BF01692917

