# Supplementary material for: Prognostic Value of the Total Bilirubin‐to‐Albumin Ratio in Critically Ill Patients with Gastrointestinal Bleeding: A Multicentre Cohort Study
Source: Emerg Med Int. 2026 Jul 25;2026:1396680. doi: 10.1155/emmi/1396680 (PMC13401164; doi:10.1155/emmi/1396680)

Supplement Table 1: Variables extracted from the MIMIC-IV database.

**items**	**composition**
**Demographic information**	Age; Gender; Race.
**Clinical severity scores**	Sepsis-Related Organ Failure Assessment Score;
**Vital signs**	Heart Rate; Systolic Blood Pressure; Diastolic Blood Pressure; Mean Arterial Pressure; Respiratory Rate; Temperature.
**Comorbidities**	Hypertension;Sepsis; Acute Kidney Injury; Cirrhosis; Diabetes; Heart Failure; Myocardial Infarction; Ischemic Heart Disease;Chronic Obstructive Pulmonary Disease;
**Laboratory test results**	Hematocrit; Hemoglobin;white blood cell;Platelets; RDW; Red Blood Cells;Anion Gap; Albumin; Total Calcium; Chloride; Glucose; Potassium; Sodium; International Normalized Ratio; prothrombin time; Activated Partial Thromboplastin Time; Alanine Aminotransferase; Aspartate Aminotransferase; Total Bilirubin; Creatinine; Blood Urea Nitrogen;Total bilirubin-to-albumin ratio
**Treatment measures**	Continuous Renal Replacement Therapy; Mechanical Ventilation,Vasopressors.
**Clinical outcome**	28d mortality;90d mortality.

Supplementary Table 2. Baseline comparison between included patients and patients excluded because of missing albumin and/or total bilirubin

**Variables**	**Included patients**<br>**(n = 1,627)**	**Excluded patients**<br>**(n = 2,338)**	**P value**
Age, years	65 (55–77)	71 (59–82)	<0.001
Gender, n (%)			0.073
Male	1,016 (62.4%)	1,394 (59.6%)	
Female	611 (37.6%)	944 (40.4%)	
Race, n (%)			0.086
White	1,052 (64.7%)	1,573 (67.3%)	
Other	575 (35.3%)	765 (32.7%)	
HR, beats/min	88 (76–99)	85 (75–96)	<0.001

Variables	Included patients (n = 1,627)	Excluded patients (n = 2,338)	P value
SBP, mmHg	112 (103–124)	116 (105–129)	<0.001
DBP, mmHg	62 (56–69)	62 (55–70)	0.859
MAP, mmHg	75 (68–83)	75 (69–83)	0.072
RR, breaths/min	18.5 (16.3–21.5)	18.5 (16.4–21.0)	0.238
Temperature, °C	36.78 (36.57–37.04)	36.77 (36.58–36.99)	0.348
Hematocrit, %	27.4 (24.5–31.0)	27.4 (24.7–31.1)	0.266
Hemoglobin, g/dL	9.08 (8.00–10.38)	9.05 (8.08–10.35)	0.472
Platelet, $\times 10^9$/L	142 (86–217)	180 (125–246)	<0.001
RDW, %	16.33 (14.90–18.30)	15.86 (14.68–17.52)	<0.001
RBC, $\times 10^{12}$/L	3.01 (2.63–3.46)	3.07 (2.71–3.53)	<0.001
WBC, $\times 10^9$/L	10.4 (7.0–15.3)	9.8 (7.1–13.7)	0.003
AG, mEq/L	14.0 (11.5–17.2)	13.5 (11.3–16.0)	<0.001
Total calcium, mg/dL	8.07 (7.60–8.58)	8.20 (7.70–8.60)	<0.001
Chloride, mEq/L	105 (100–109)	106 (102–110)	<0.001
Glucose, mg/dL	128 (106–162)	121 (101–152)	<0.001
Potassium, mEq/L	4.20 (3.80–4.65)	4.10 (3.80–4.50)	<0.001
Sodium, mEq/L	138.5 (135.0–141.5)	139.3 (136.5–142.0)	<0.001
INR	1.45 (1.20–1.80)	1.30 (1.14–1.57)	<0.001
PT, s	15.8 (13.6–19.8)	14.2 (12.7–17.0)	<0.001
APTT, s	33 (28–42)	30 (27–36)	<0.001
ALT, IU/L	26 (15–54)	24 (15–47)	0.147
AST, IU/L	45 (23–102)	39 (22–84)	0.007
Creatinine, mg/dL	1.23 (0.80–2.20)	1.10 (0.80–1.80)	<0.001
BUN, mg/dL	32 (19–53)	29 (18–49)	<0.001
SOFA	6.0 (4.0–10.0)	4.0 (2.0–7.0)	<0.001
HTN, n (%)	530 (32.6%)	882 (37.7%)	<0.001
AKI, n (%)	862 (53.0%)	1,005 (43.0%)	<0.001
Cirrhosis, n (%)	557 (34.2%)	330 (14.1%)	<0.001
Diabetes, n (%)	469 (28.8%)	728 (31.1%)	0.119
HF, n (%)	482 (29.6%)	770 (32.9%)	0.027
MI, n (%)	135 (8.3%)	191 (8.2%)	0.885
IHD, n (%)	510 (31.3%)	884 (37.8%)	<0.001
COPD, n (%)	220 (13.5%)	345 (14.8%)	0.274
Sepsis, n (%)	1,101 (67.7%)	1,126 (48.2%)	<0.001
CRRT, n (%)	193 (11.9%)	138 (5.9%)	<0.001
MV, n (%)	1,281 (78.7%)	1,587 (67.9%)	<0.001
Vasopressors, n (%)	851 (52.3%)	861 (36.8%)	<0.001
28-day mortality, n (%)	295 (18.1%)	270 (11.5%)	<0.001

Variables	Included patients (n = 1,627)	Excluded patients (n = 2,338)	P value
90-day mortality, n (%)	370 (22.7%)	355 (15.2%)	<0.001

Continuous variables are presented as median (interquartile range, 25th–75th percentile), and categorical variables are presented as n (%). P values were calculated using the Mann–Whitney U test for continuous variables and the chi-square test or Fisher's exact test for categorical variables, as appropriate. Abbreviations: HR, heart rate; SBP, systolic blood pressure; DBP, diastolic blood pressure; MAP, mean arterial pressure; RR, respiratory rate; RDW, red blood cell distribution width; RBC, red blood cell; WBC, white blood cell; AG, anion gap; INR, international normalized ratio; PT, prothrombin time; APTT, activated partial thromboplastin time; ALT, alanine aminotransferase; AST, aspartate aminotransferase; BUN, blood urea nitrogen; SOFA, Sequential Organ Failure Assessment; HTN, hypertension; AKI, acute kidney injury; HF, heart failure; MI, myocardial infarction; IHD, ischemic heart disease; COPD, chronic obstructive pulmonary disease; CRRT, continuous renal replacement therapy; MV, mechanical ventilation.

Supplementary Table 3. Sensitivity analysis after multiple imputation for missing serum albumin and total bilirubin: association of TBAR with 28-day and 90-day mortality.

Variable	Model 1		Model 2		Model 3	
	HR (95% CI)	P value	HR (95% CI)	P value	HR (95% CI)	P value
**28-day mortality**						
TBAR (continuous)	1.22 (1.19–1.25)	<0.001	1.18 (1.15–1.21)	<0.001	1.12 (1.09–1.16)	<0.001
**TBAR (categorical)**						
Low TBAR	1		1		1	
High TBAR	4.60 (3.79–5.58)	<0.001	3.83 (3.14–4.69)	<0.001	2.32 (1.85–2.90)	<0.001
**90-day mortality**						
TBAR (continuous)	1.21 (1.18–1.23)	<0.001	1.17 (1.14–1.20)	<0.001	1.12 (1.09–1.15)	<0.001
**TBAR (categorical)**						
Low TBAR	1		1		1	
High TBAR	3.94 (3.30–4.70)	<0.001	3.35 (2.79–4.03)	<0.001	2.10 (1.71–2.59)	<0.001

Values are presented as hazard ratios (HRs) with 95% confidence intervals (CIs). The analysis included 3,965 patients after imputation. TBAR was recalculated after imputation of missing serum albumin and total bilirubin values.

Model 1: adjusted for age, gender, and race.

Model 2: adjusted for age, gender, race, heart rate, respiratory rate, red blood cell count, and anion gap.

Model 3: adjusted for age, gender, race, heart rate, respiratory rate, red blood cell count, anion gap, activated partial thromboplastin time, blood urea nitrogen, SOFA score, acute kidney injury, cirrhosis, diabetes mellitus, sepsis, continuous renal replacement therapy, mechanical ventilation, and vasopressor use.

Supplementary Table 4. Risk stratification according to nomogram-derived predicted probabilities of 28-day mortality.

Risk group	Nomogram-derived predicted probability	Patients, n (%)	28-day deaths, n	Observed 28-day mortality, %	Mean predicted risk, %	P value
Low risk	<10%	728 (44.7)	31	4.3	5.3	<0.001
Intermediate risk	10%–20%	360 (22.1)	63	17.5	14.6	
High risk	≥20%	539 (33.1)	201	37.3	37.8	

Values are presented as n (%) unless otherwise specified. The derivation cohort included 1,627 patients, of whom 295 died within 28 days after ICU admission.

Risk groups were defined according to nomogram-derived predicted probabilities of 28-day mortality: low risk (<10%), intermediate risk (10%–20%), and high risk (≥20%).

Supplementary Table 5. External validation in the eICU-CRD cohort.

Variable	Model 1		Model 2		Model 3	
	HR (95% CI)	P value	HR (95% CI)	P value	HR (95% CI)	P value
**eICU-CRD mortality**						
TBAR (continuous)	1.18 (1.15–1.21)	<0.001	1.15 (1.12–1.18)	<0.001	1.07 (1.03–1.11)	<0.001
**TBAR (binary)**						
TBAR ≤2.05	1		1		1	
TBAR >2.05	3.94 (3.08–5.04)	<0.001	2.92 (2.24–3.80)	<0.001	1.39 (1.04–1.86)	0.026
**Sensitivity analysis around the TBAR cutoff**						
TBAR ≤1.8	1		1		1	
TBAR >1.8	4.07 (3.21–5.15)	<0.001	3.15 (2.44–4.07)	<0.001	1.57 (1.18–2.09)	0.002
TBAR ≤2.2	1		1		1	
TBAR >2.2	4.17 (3.25–5.34)	<0.001	3.10 (2.38–4.05)	<0.001	1.55 (1.17–2.06)	0.003
**TBAR (quartiles)**						
Q1 [0.0256–0.189)	1		1		1	
Q2 [0.189–0.333)	1.85 (1.24–2.76)	0.003	1.81 (1.22–2.70)	0.004	1.50 (1.00–2.24)	0.049
Q3 [0.333–0.692)	3.01 (2.08–4.37)	<0.001	2.90 (2.00–4.22)	<0.001	1.93 (1.32–2.83)	<0.001
Q4 [0.692–20.2]	6.28 (4.39–8.98)	<0.001	5.32 (3.70–7.64)	<0.001	2.04 (1.37–3.06)	<0.001
P for trend		<0.001		<0.001		<0.001

Values are presented as hazard ratios (HRs) with 95% confidence intervals (CIs).

Model 1: adjusted for age, gender, and race.

Model 2: adjusted for age, gender, race, HR, RR, RBC, and AG.

Model 3: adjusted for age, gender, race, HR, RR, RBC, AG, APTT, BUN, SOFA, AKI, cirrhosis, diabetes mellitus, sepsis, CRRT, MV, and vasopressors.

HR, hazard ratio; CI, confidence interval; TBAR, total bilirubin-to-albumin ratio; eICU-CRD, electronic Intensive Care Unit Collaborative Research Database.

Supplementary Fig 1: Ten-fold cross-validation for LASSO model tuning.

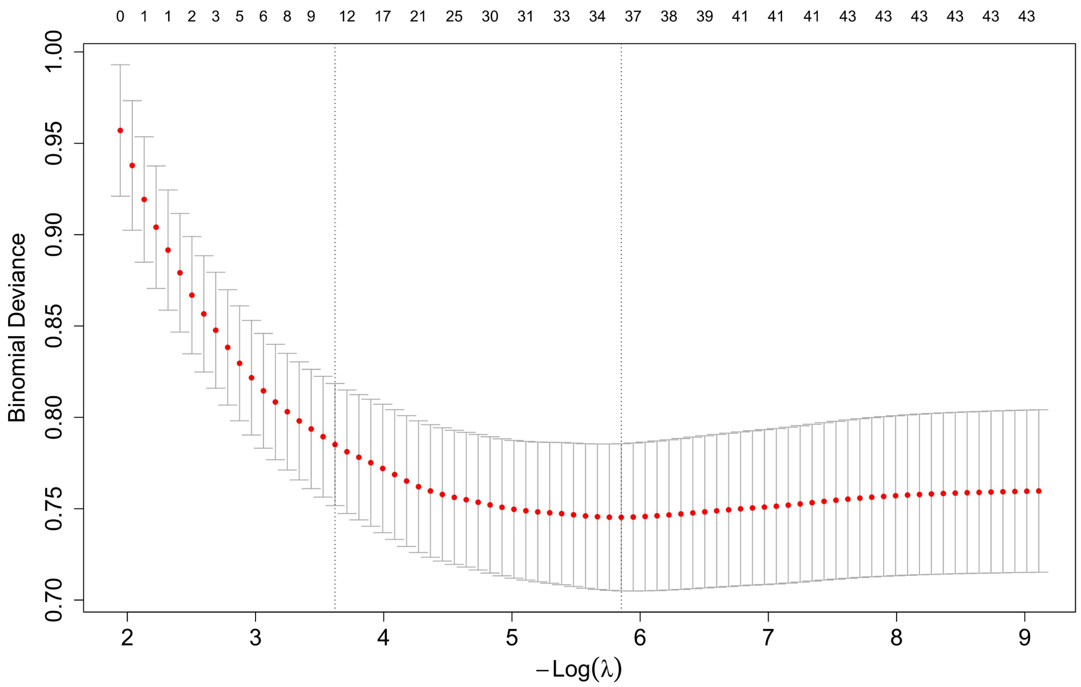

Supplementary Fig 2: LASSO coefficient profiles of candidate predictors.

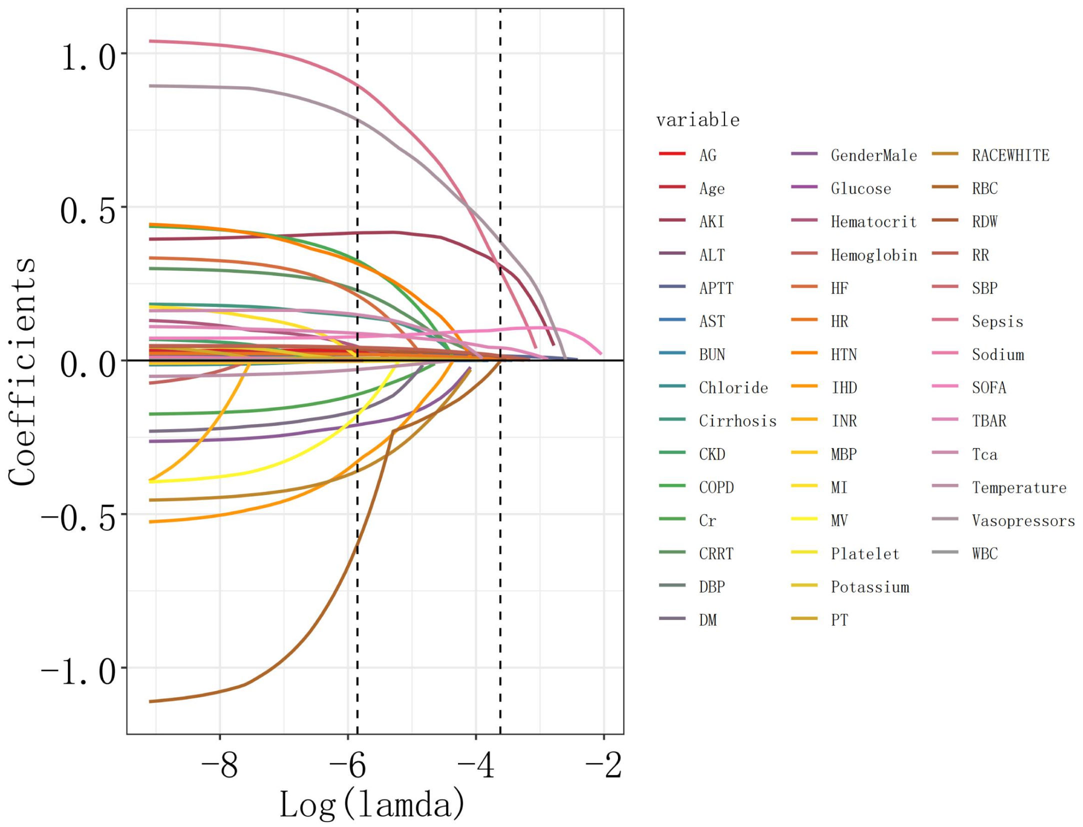

Supplementary Fig 3: Optimal λ selection and identification of final features.

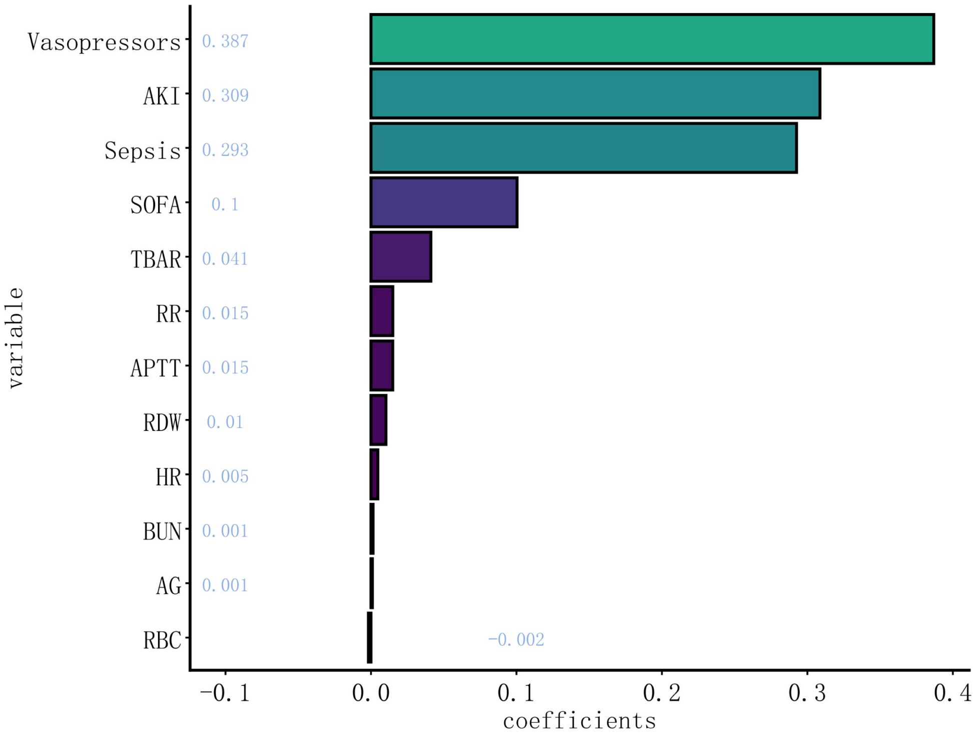

Supplementary Fig 4: Boruta-selected features for 28-day mortality.

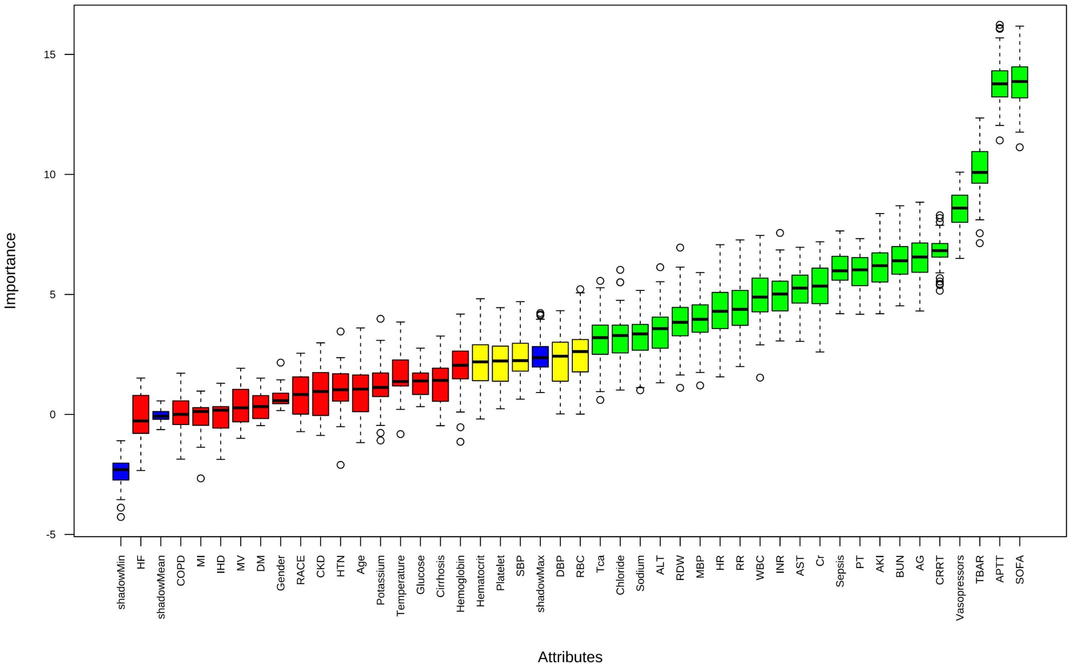

Supplementary Fig 5: ROC curve showing the discriminative performance of the nomogram for 28-day mortality.

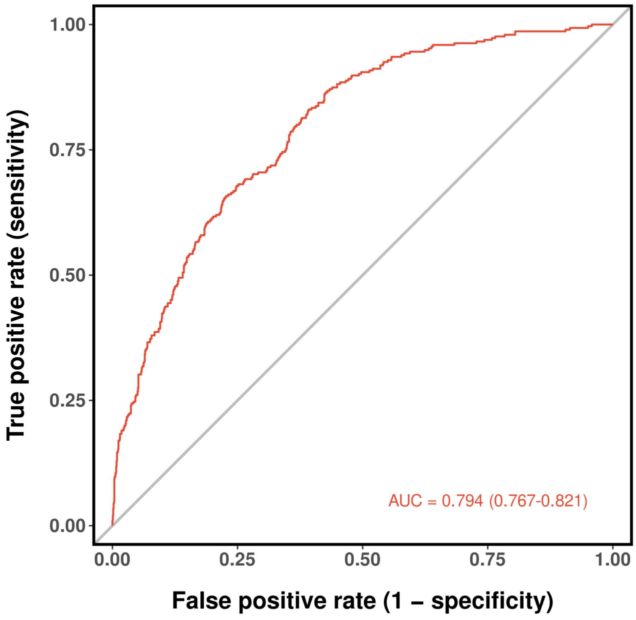

Supplementary Fig 6: Calibration plot illustrating the agreement between predicted and observed 28-day mortality.

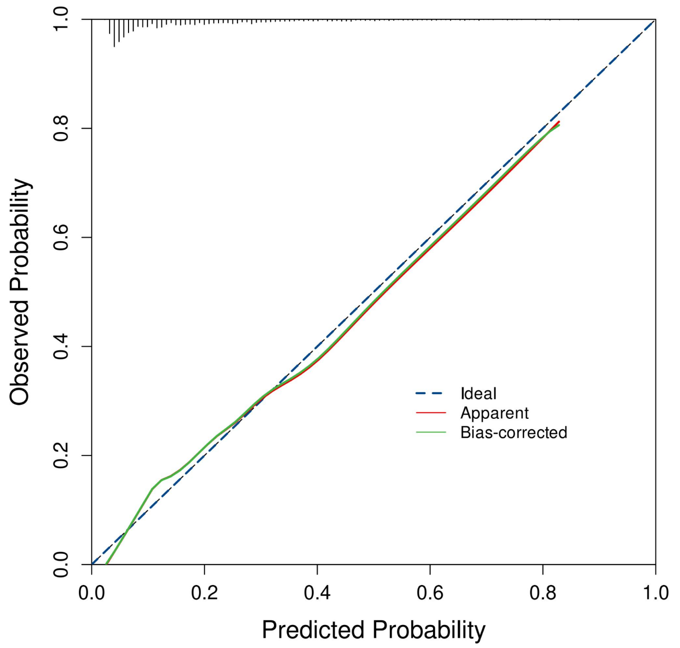

Supplementary Fig 7: Decision curve analysis for 28-day mortality.

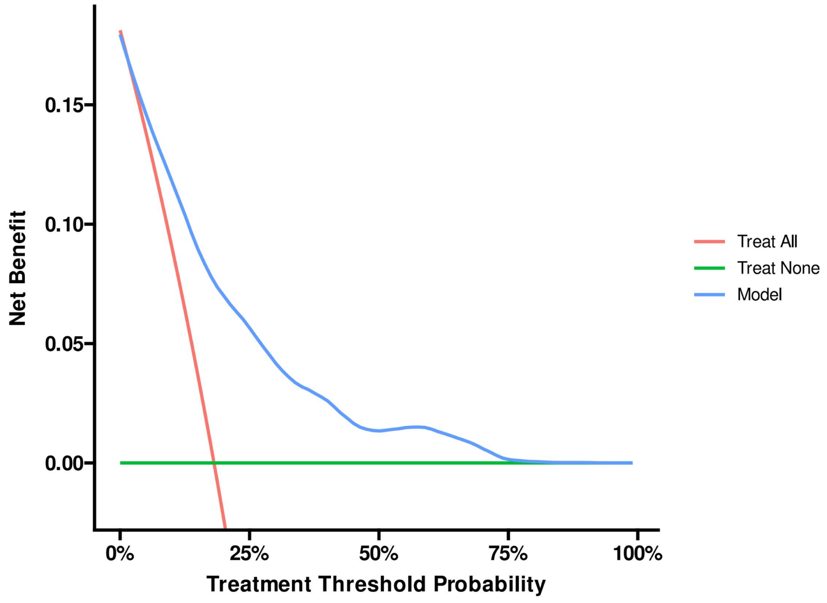

Supplementary Fig 8: K–M survival curves stratified by TBAR levels in external validation.

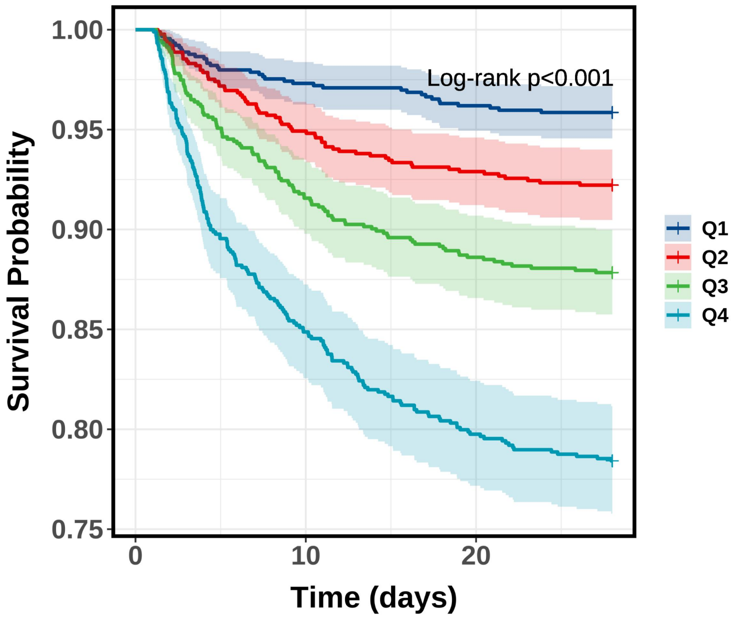

Supplementary Fig 9: External validation ROC curve using the eICU-CRD cohort.

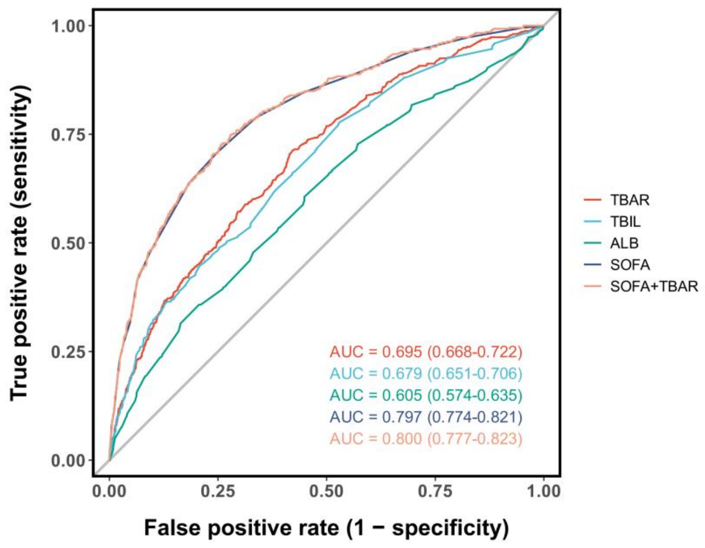

Supplementary Fig 10: Restricted cubic spline of TBAR in the eICU-CRD cohort.

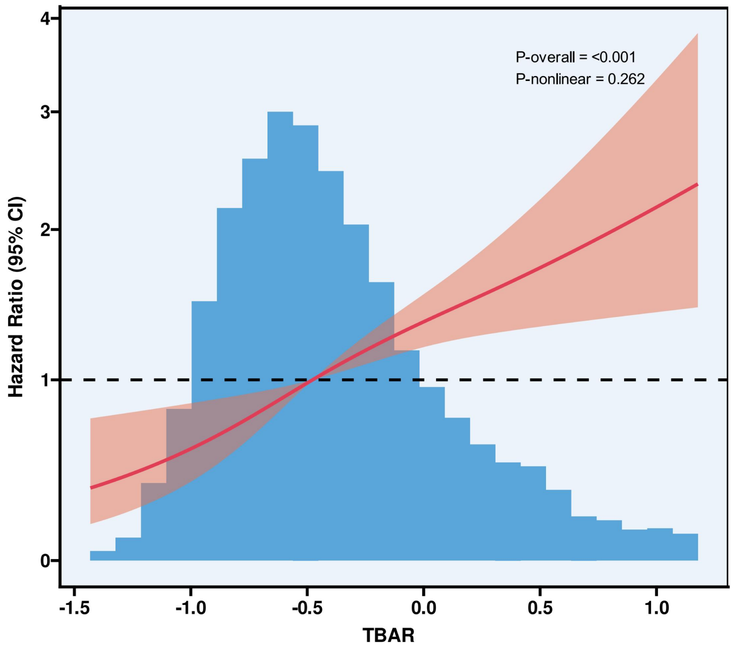

Supplementary Fig 11: ROC analysis of the nomogram for external validation

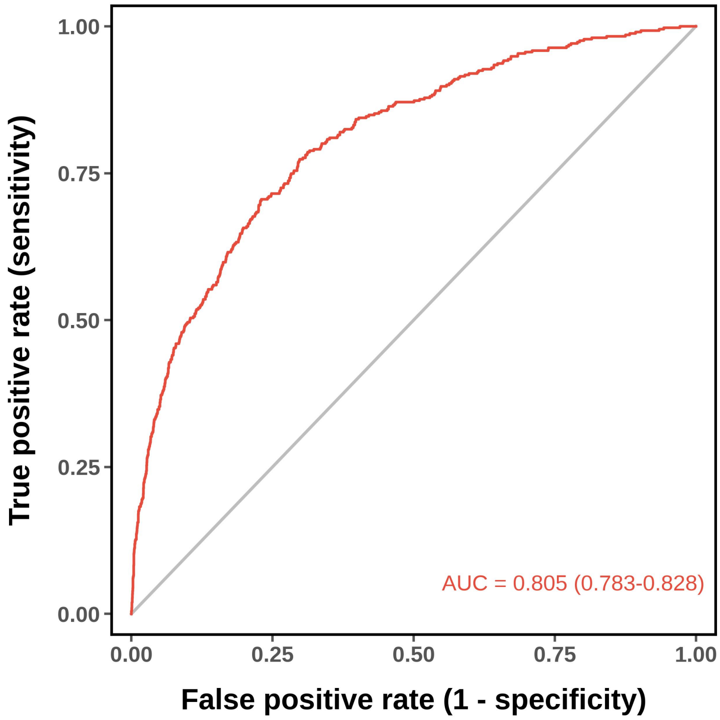

Supplement: Supplementary file 1 — Supporting Information 1 Supporting Table 1: Variables extracted from the MIMIC‐IV database. Supporting Table 2: Baseline comparison between included patients and patients excluded because of missing albumin and/or total bilirubin. Supporting Table 3: Sensitivity analysis after multiple imputation for missing serum albumin and total bilirubin: association of TBAR with 28‐day and 90‐day mortality. Supporting Table 4: Risk stratification according to nomogram‐derived predicted probabilities of 28‐day mortality. Supporting Table 5: External validation in the eICU‐CRD cohort. Supporting Figure 1: Ten‐fold cross‐validation for LASSO model tuning. Supporting Figure 2: LASSO coefficient profiles of candidate predictors. Supporting Figure 3: Optimal λ selection and identification of final features. Supporting Figure 4: Boruta‐selected features for 28‐day mortality. Supporting Figure 5: ROC curve showing the discriminative performance of the nomogram for 28‐day mortality. Supporting Figure 6: Calibration plot illustrating the agreement between predicted and observed 28‐day mortality. Supporting Figure 7: Decision curve analysis for 28‐day mortality. Supporting Figure 8: K–M survival curves stratified by TBAR levels in external validation. Supporting Figure 9: External validation ROC curve using the eICU‐CRD cohort. Supporting Figure 10: Restricted cubic spline of TBAR in the eICU‐CRD cohort. Supporting Figure 11: ROC analysis of the nomogram for external validation. [file EMMI-2026-1396680-s001.pdf]
